# Book review

**Published:** 2013

**Figure:**
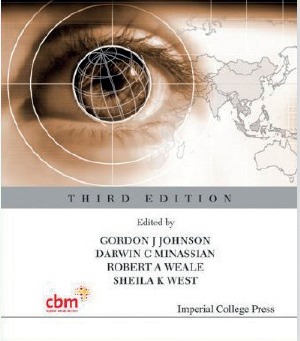
The Epidemiology of Eye Disease, 3rd Edition, edited by Johnson GJ et al

The third edition of this authoritative text is 240 pages longer than the previous version and has 15 additional expert contributors. There is an extra chapter on research synthesis and an expanded section on visual impairment and blindness in children. Dry eye and uveitis now feature as separate entities, and the final two chapters address the practical application of epidemiology in changing people's lives for the better. (Reviewed by Nick Astbury.)

**Cost**: UK £98. Readers can quote

**WSOPH** to get a 50percnt; discount when ordering from: World Scientific Publishing (UK) Ltd., c/o Marston Book Services, PO Box 269, Abingdon, Oxon OX14 4YN, UK. Email: direct.orders@marston.co.uk

## Subscriptions

Would you like to receive your own copy of the *Community Eye Health Journal?* Or have you changed address? Send your name, occupation, email address and home address to: Anita Shah, International Centre for Eye Health, London School of Hygiene and Tropical Medicine, London WC1E 7HT, UK. Email: admin@cehjournal.org

**Figure:**
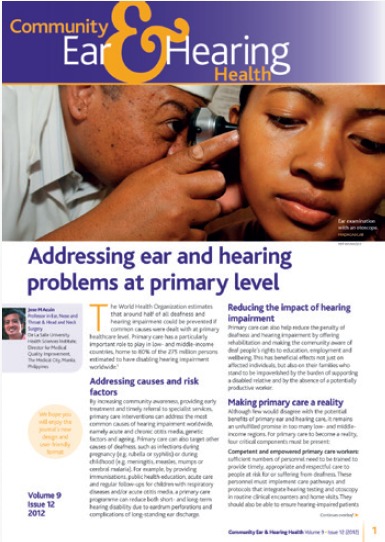
Community Ear and Hearing Health

Like the *Community Eye Health Journal*, this journal is sent free of charge to readers from low-and middle-income countries. It aims to promote ear and hearing health by offering continuing education for all levels of health worker. To subscribe, please email: Joanna.Anderson@Lshtm.ac.uk

## Courses

### Community Eye Health Institute, University of Cape Town, South Africa

Contact: Zanele Magwa, Community Eye Health Institute, University of Cape Town, Private Bag 3, Rondebosch 7700, South Africa. Tel: +27 21 404 7735. Email: ntombizanele.magwa@uct.ac.za

### Kilimanjaro Centre for Community ophthalmology (KCCO), Tanzania

For information on courses, contact Genes Mng'anya, KCCO Tanzania Limited PO Box 2254, Moshi, Tanzania. Tel: +255 27 275 3547. Visit www.kcco.net

### Lions SightFirst Eye Hospital, Nairobi, Kenya

**Small incision cataract surgery for ophthalmologists wishing to upgrade from ECCE**. Write to: The Training Coordinator, Lions Medical Training Centre, Lions SightFirst Eye Hospital, PO Box 66576–00800, Nairobi, Kenya. Tel: +254 20 418 32 39. Email: training@lionsloresho.org

